# Relationship between Lipids Levels of Serum and Seminal Plasma and Semen Parameters in 631 Chinese Subfertile Men

**DOI:** 10.1371/journal.pone.0146304

**Published:** 2016-01-04

**Authors:** Jin-Chun Lu, Jun Jing, Qi Yao, Kai Fan, Guo-Hong Wang, Rui-Xiang Feng, Yuan-Jiao Liang, Li Chen, Yi-Feng Ge, Bing Yao

**Affiliations:** 1 The Reproductive Medical Center, Nanjing Jinling Hospital, Nanjing University School of Medicine, Nanjing, Jiangsu, China; 2 Department of Laboratory Science, Nanjing Hospital, Jiangsu Corps, The Armed Police Force, PLA, Nanjing, Jiangsu, China; Nanjing Medical University, CHINA

## Abstract

**Objective:**

This prospective study was designed to investigate the relationship between lipids levels in both serum and seminal plasma and semen parameters.

**Methods:**

631 subfertile men were enrolled. Their obesity-associated markers were measured, and semen parameters were analyzed. Also, seminal plasma and serum TC, TG, HDL and LDL and serum FFA, FSH, LH, total testosterone (TT), estradiol (E2) and SHBG levels were detected.

**Results:**

Seminal plasma and serum TG, TC and LDL levels were positively related to age. Serum TC, TG and LDL were positively related to obesity-associated markers (*P* < 0.001), while only seminal plasma TG was positively related to them (*P* < 0.05). For lipids levels in serum and seminal plasma, only TG level had slightly positive correlation between them (*r* = 0.081, *P* = 0.042). There was no significant correlation between serum lipids levels and semen parameters. However, seminal plasma TG, TC, LDL and HDL levels were negatively related to one or several semen parameters, including semen volume (SV), sperm concentration (SC), total sperm count (TSC), sperm motility, progressive motility (PR) and total normal-progressively motile sperm counts (TNPMS). Moreover, seminal plasma TG, TC, LDL and HDL levels in patients with oligospermatism, asthenospermia and teratozoospermia were higher than those with normal sperm concentration, motility or morphology. After adjusting age and serum LH, FSH, TT, E2 and SHBG levels, linear regression analysis showed that SV was still significantly correlated with seminal plasma LDL (*P* = 0.012), both of SC and TSC with seminal plasma HDL (*P* = 0.028 and 0.002), and both of PR and sperm motility with seminal plasma TC (*P* = 0.012 and 0.051).

**Conclusion:**

The abnormal metabolism of lipids in male reproductive system may contribute to male factor infertility.

## Introduction

Numerous studies indicated that obesity is closely associated with higher prevelence of male factor infertility [1–4]. Increased hypertension, 4hyperlipidemia and metabolism syndrome are major factors contributing to obesity [5–6]. Among them, abnormal lipid metabolism was closely associated with the disorder of spermatogenesis, sperm maturation and capacitation [7–9]. Lu *et al* [10] previously showed that obesity-associated markers such as body mass index (BMI), waist circumference (WC), waist-to-hip ratio (WHR) and waist-to-height ratio (WHtR) could not predict semen quality. However, it was unclear whether lipids levels in serum and seminal plasma reflect semen quality. Therefore, we designed this study to investigate the correlations between lipids levels, including total cholesterol (TC), triglyceride (TG), high density lipoprotein (HDL), low density lipoprotein (LDL) and free fatty acid (FFA), in serum and seminal plasma, and obesity-associated markers such as BMI, WC, WHR and WHtR, and between lipids levels in serum and seminal plasma and semen parameters in 631 subfertile men.

## Materials and Methods

### Study population

This is a prospective study. Male subfertile patients, aged from 18 to 55 years with female partners stopping use of contraception in order to try to conceive for over 12 months, who attended the out-patient clinic at Nanjing Jingling Hospital for diagnosis of infertility between August 2012 and February 2015, were included in this study. All participants were asked to complete a questionnaire to collect basic information on occupation, medical and reproductive history and lifestyle factors including intakes of alcohol and tobacco smoking. Then, all participants underwent physical examination, and obesity-associated markers were measured, semen samples were collected by masturbation, and venous blood samples after 8–12 hours of fasting were drawn during 8: 00 am and 10: 00 am. Stringent exclusion criteria were employed to exclude regular alcohol drinkers, heavy smokers, the men with chronic diseases, urogenital infections, varicocele and other diseases which might lead to dysspermia, azoospermic men, and the men with 100% of immotile sperm, 100% teratospermia and incomplete data. Six hundred and thirty-one (631) men were enrolled in this study. This study was approved by the Human Subject Committees of Nanjing Jinling Hospital, and informed consent was signed by each participant.

### Measurement of obesity-associated markers

Height and weight were measured with each participant standing without shoes and heavy outer garments. WC was measured at the level midway between the lower rib margin and the iliac crest with participant in standing position without heavy outer garments and with emptied pockets, breathing out gently. Hip circumference was recorded as the maximum circumference over the buttocks. BMI was calculated as weight divided by height squared (kg/m^2^). WHR was calculated as the ratio of WC over the hip circumference. WHtR was calculated as the ratio of WC over height.

### Determination of serum lipids

Venous blood samples were centrifuged at 3 000 g for 5 min to isolate serum for detection of lipids level. Commercially available kits for the determinations of TC, TG, HDL and LDL, and calibration and quality control products were purchased from Randox Laboratories Ltd., Northern Ireland, United Kingdom. The determinations were carried out using Hitachi 7600–210 automatic biochemical analyzer. Commercially available kit for the determination of FFA was purchased from Nanjing Jiancheng Bioengineering Institute (Jiangsu, China). The determinations of TC, TG, HDL, LDL and FFA were performed strictly according to the manufacturer’s instruction.

### Analysis of semen parameters

Semen samples were collected by masturbation after sexual abstinence for 2–7 days and kept at 37°C for 30 min. After liquefaction, each semen sample volume was measured by weighing, followed by routine analysis, and the remaining semen samples were centrifuged at 12 000 g for 5 min. The upper layer seminal plasma was collected for the determination of lipids. Sperm concentration, total motility and progressive motility (PR) were analyzed by computer-aided sperm analysis (CASA) system with phase contrast microscope (CFT-9201, Jiangsu Rich Life Science Instrument Co., Ltd., Nanjing, China) [11], and sperm morphology was evaluated using Diff-Quik staining. For each specimen, at least 200 spermatozoa were analyzed for each replicate. When difference between two replicates was within an acceptable range (95% confidence interval), the average value was calculated and documented. When difference was higher, the assessment was repeated by taking two new aliquots from the same semen sample [12]. Then, a new parameter—total normal-progressively motile sperm count (semen volume × sperm concentration × progressive motility × percent of normal sperm morphology, TNPMS) was calculated, because the determinative factor for male fertility is the spermatozoa with motility and normal morphology. The criteria for oligozoospermia, asthenospermia and teratozoospermia were in accordance with the World Health Organization guidelines [12].

### Determination of lipids in seminal plasma

Commercially available kits for the determinations of TG, TC, LDL and HDL were purchased from Shanghai Zhicheng Biotechnology Co., Ltd., China. Calibration and quality control products were purchased from Randox Laboratories Ltd., Northern Ireland, United Kingdom. Determination of lipids in seminal plasma was carried out using Olympus AU400 automatic biochemistry analyzer (Olympus Optical Co. Ltd., Japan). The sample with higher lipid level exceeding the linear range of the kit should be diluted with normal saline and the diluted volume was calculated.

### Determination of serum reproductive hormones

A non-fasting blood sample was drawn the same day that the semen sample was produced. Blood was centrifuged and serum was stored at −80°C until analysis. Sera were then thawed and analyzed for total testosterone (TT), luteinizing hormone (LH), follicle-stimulating hormone (FSH), estradiol (E2), and sex hormone-binding globulin (SHBG) levels. TT, LH, FSH, E2 and SHBG levels were determined by chemiluminescence assay using an automated Unicel Dxi 800 Access Immunoassay System (Beckman Coulter, Inc., USA). The assay of serum TT and E2 was based on a competitive principle using a high affinity monoclonal antibody (sheep) specifically directed against testosterone or E2. The assay of serum LH, FSH and SHBG was based on a sandwich principle using two different monoclonal antibodies specifically directed against human LH, FSH or SHBG. The assay sensitivities were 0.35 nmol/L for TT, 0.2 IU/L for LH, 0.2 IU/L for FSH, 73 pmol/L for E2 and 0.33 nmol/L for SHBG. The intra-assay coefficients of variation (CV) for LH, FSH, TT, E2 and SHBG were all less than 5%, and the inter-assay CVs were all less than 8%.

### Statistical analysis

All data were analyzed using SPSS 11.0 software (SPSS Inc., Chicago, IL, USA). First, nonparametric tests (one-sample Kolmogorov-Smirnov test) were used to determine whether parameters were consistent with normal distribution. Correlations between and within lipids levels in serum and seminal plasma, obesity-associated markers and semen parameters were examined by Pearson test, while the parameters were inconsistent with normal distribution, correlations were examined by Spearman’s rho test. Differences between categories were assessed by independent-samples *t*-test. The effects of age, reproductive hormones and seminal plasma lipids on semen parameters were analyzed by linear regression. *P*-value ≤ 0.05 was considered statistically significant.

## Results

### General data

Among 631 subfertile men, 81 with sperm concentration < 15×10^6^/ml, 58 with total sperm count < 39×10^6^/ejaculate, 285 with PR < 32%, 231 with sperm motility < 40%, 183 with normal sperm morphology < 4%, and 248 with normal sperm concentration, PR, motility and morphology were detected. Among 631 subfertile men, there were 202, 162, 249 and 259 men with serum TG, TC, LDL and HDL levels out of their normal reference values (0.2–1.71 mmol/L for TG, 3.25–5.2 mmol/L for TC, 2–3.12 mmol/L for LDL and 1.1–1.55 mmol/L for HDL), respectively, and 147 with serum TG, TC, LDL and HDL levels all within their normal reference values. In addition, serum reproductive hormones levels from 594 of 631 subfertile men were determined. Table 1 summarized the data including age, obesity-associated markers, semen parameters, lipids levels in serum and seminal plasma, and serum reproductive hormones for 631 subfertile men.

**Table 1:** Mean and range of age, obesity-associated markers, serum and seminal plasma lipids levels, serum reproductive hormones levels and semen parameters in 631 subfertile men.

Variable	*n*	Mean (SD)	Range
**Age (years)**	631	29.37 (4.48)	19–50
**BMI (kg/m**^**2**^**)**	631	24.13 (3.08)	17.47–41.03
**WC (cm)**	631	82.99 (9.30)	60–131
**WHtR**	631	0.48 (0.052)	0.34–0.74
**WHR**	631	0.86 (0.058)	0.70–1.13
**Semen volume (ml)**	631	3.64 (1.32)	1.50–9.50
**Sperm concentration (10**^**6**^**/ml)**	631	57.15 (44.67)	0.67–242.09
**Total sperm count (10**^**6**^**/ejaculate)**	631	196.22 (158.23)	1.35–1127.70
**Progressive motility (%)**	631	33.14 (13.06)	0.91–74.10
**Sperm motility (%)**	631	45.61 (18.42)	1.51–90.69
**Normal sperm morphology (%)**	631	4.31 (1.69)	0.42–9.05
**TNPMS (10**^**6**^**/ejaculate)**	631	3.06 (3.10)	0.01–23.92
**Serum TG (mmol/L)**	631	1.75 (1.67)	0.05–23.34
**Serum TC (mmol/L)**	631	4.54 (1.00)	0.95–12.19
**Serum LDL (mmol/L)**	631	2.55 (0.74)	0.02–5.43
**Serum HDL (mmol/L)**	631	1.26 (0.29)	0.65–3.04
**Serum FFA (μmol/L)**	631	725.43 (511.26)	32.26–5612.90
**Seminal plasma TG (mmol/L)**	631	0.11 (0.09)	0.01–0.90
**Seminal plasma TC (mmol/L)**	631	0.82 (0.50)	0.05–3.11
**Seminal plasma LDL (mmol/L)**	631	0.70 (0.32)	0.08–2.08
**Seminal plasma HDL (mmol/L)**	631	0.37 (0.20)	0.01–1.31
**Serum LH (IU/L)**	594	3.98 (1.62)	0.62–11.89
**Serum FSH (IU/L)**	594	4.83 (2.31)	1.11–18.17
**Serum TT (nmol/L)**	594	13.09 (3.94)	3.68–31.93
**Serum E2 (pmol/L)**	594	107.54 (50.40)	18.00–331.00
**Serum SHBG (nmol/L)**	594	26.21 (10.73)	6.20–69.40

TNPMS, total normal-progressively motile sperm count.

### Correlations of serum lipids levels with age, obesity-associated markers and semen parameters

Variables, including serum lipids levels, age, obesity-associated markers and semen parameters in 631 subfertile men, were inconsistent with normal distribution, therefore, correlations between these variables were analyzed by Spearman’s rho test. Table 2 showed the results. Serum TG, TC and LDL levels were positively related to age (*P* < 0.001). Serum TC, TG and LDL levels were positively related to BMI, WC, WHtR and WHR (*P* < 0.001), while serum HDL level was negatively related to BMI (*P* < 0.001). There was no correlation between serum FFA level and these obesity-associated markers. Serum TC, TG, LDL, HDL and FFA levels were not related to semen parameters including semen volume, sperm concentration, total sperm count, progressive motility, total motility, normal sperm morphology and TNPMS. However, the data showed slightly negative correlations between serum TG level and semen volume (*P* = 0.045), and between serum FFA level and sperm motility (*P* = 0.048), and slightly positive correlation between serum HDL level and semen volume (*P* = 0.013). In addition, significantly positive correlations were found between serum TC, TG and LDL levels (*P* < 0.001), between serum TC and LDL levels and HDL level, and between serum HDL and FFA levels (*P* = 0.006), while negative correlation between serum TG level and HDL and FFA levels (*P* < 0.001).

**Table 2:** Nonparametric (Spearman) correlation coefficients between serum or seminal plasma lipids levels and age, obesity-associated markers and semen parameters.

Variable	Serum TG	Serum TC	Serum LDL	Serum HDL	Serum FFA	TG in SP	TC in SP	LDL in SP	HDL in SP
**Age**	0.193^a^	0.255^a^	0.225^a^	-0.057	-0.004	0.163^a^	0.145^a^	0.120^b^	0.067
**BMI**	0.375^a^	0.205^a^	0.235^a^	-0.278^a^	0.022	0.079^b^	0.030	0.058	0.039
**WC**	0.499^a^	0.297^a^	0.296^a^	-0.301^a^	-0.049	0.119^b^	0.000	0.074	-0.018
**WHtR**	0.498^a^	0.317^a^	0.323^a^	-0.305^a^	-0.034	0.121^b^	-0.009	0.058	-0.014
**WHR**	0.472^a^	0.296^a^	0.279^a^	-0.301^a^	-0.060	0.083^b^	0.022	0.088b	0.032
**Semen volume**	-0.080^b^	-0.073	-0.044	0.099^b^	0.061	-0.068	-0.022	-0.129^a^	-0.045
**SC**	-0.011	0.026	0.020	0.055	-0.002	-0.018	-0.056	0.025	-0.114^b^
**TSC**	-0.023	-0.009	-0.004	0.076	0.030	-0.029	-0.055	-0.018	-0.135^a^
**PR**	-0.019	0.021	0.040	0.042	-0.062	-0.080^b^	-0.094^b^	-0.090^b^	0.058
**MOT**	-0.035	0.015	0.036	0.044	-0.079^b^	-0.043	-0.091^b^	-0.066	0.013
**NSM**	0.026	-0.012	0.016	0.003	-0.043	0.028	-0.045	0.000	-0.018
**TNPMS**	-0.021	0.007	0.028	0.069	-0.025	-0.038	-0.093^b^	-0.053	-0.077
**Serum TG**	-	0.454^a^	0.318^a^	-0.338^a^	-0.147^a^	-	-	-	-
**Serum TC**	-	-	0.826^a^	0.209^a^	-0.028	-	-	-	-
**Serum LDL**	-	-	-	0.088^b^	-0.014	-	-	-	-
**Serum HDL**	-	-	-	-	0.109^b^	-	-	-	-
**TG in SP**	-	-	-	-	-	-	0.580^a^	0.432^a^	0.216^a^
**TC in SP**	-	-	-	-	-	-	-	0.795^a^	0.648^a^
**LDL in SP**	-	-	-	-	-	-	-	-	0.481^a^

SC, sperm concentration; TSC, total sperm count; PR, progressive motility; MOT, sperm motility; NSM, normal sperm morphology; TNPMS, total normal-progressively motile sperm count; SP, seminal plasma.

^a^, *P* ≤ 0.001

^b^, *P* ≤ 0.05

Serum TG, TC and LDL levels were positively related to age. Serum TC, TG and LDL levels were positively related to BMI, WC, WHtR and WHR, while serum HDL level was negatively related to them. There were slightly negative correlations between serum TG level and semen volume (*P* = 0.045), and between serum FFA level and sperm motility (*P* = 0.048), and slightly positive correlation between serum HDL level and semen volume (*P* = 0.013). There were significantly positive correlations between serum TC, TG and LDL levels, between serum TC and LDL levels and HDL level, and between serum HDL and FFA levels, while negative correlation between serum TG level and HDL and FFA levels. TG, TC and LDL levels in SP were positively related to age. TG level in SP was positively related to BMI, WC, WHtR and WHR, while TC, LDL and HDL levels in SP were not related to them except slightly positive correlation between LDL level and WHR. TG level in SP was negatively related to PR; TC level was negatively related to PR, sperm motility and TNPMS; LDL level was negatively related to semen volume and PR; HDL level was negatively related to sperm concentration and total sperm count. There were positive correlations between TG, TC, LDL and HDL levels in SP.

### Correlations of lipids levels in seminal plasma with age, obesity-associated markers and semen parameters

Variables, including lipids levels in seminal plasma, age, obesity-associated markers and semen parameters in 631 subfertile men, were inconsistent with normal distribution, thus, correlations between these variables were analyzed by Spearman’s rho test, and the results were also shown in Table 2. TG, TC and LDL levels in seminal plasma were positively related to age (*P* < 0.05). Seminal plasma TG level was positively related to BMI, WC, WHtR and WHR (*P* = 0.046, 0.003, 0.002 and 0.038, respectively), while TC, LDL and HDL levels were unrelated to them except slightly positive correlation between LDL level and WHR (*P* = 0.027). Analysis on the correlations between lipids levels in seminal plasma and semen parameters showed that TG level was negatively related to PR (*r* = -0.080, *P* = 0.045), TC level was negatively related to PR (*r* = -0.094, *P* = 0.018), sperm motility (*r* = -0.091, *P* = 0.023) and TNPMS (*r* = -0.093, *P* = 0.020), LDL level was negatively related to semen volume (*r* = -0.129, *P* = 0.001) and PR (*r* = -0.090, *P* = 0.024), and HDL level was negatively related to sperm concentration (*r* = -0.114, *P* = 0.004) and total sperm count (*r* = -0.135, *P* = 0.001). In addition, there were positive correlations between TG, TC, LDL and HDL levels in seminal plasma.

### Relationship between lipids levels in serum and seminal plasma

Table 3 summarized nonparametric (Spearman) correlation coefficients for the relationship between lipids levels in serum and seminal plasma in 631 subfertile men. There was no correlation between lipids levels in serum and seminal plasma except slightly positive correlation of TG level in seminal plasma with serum TG and LDL levels (*P* = 0.042 and 0.026).

**Table 3:** Nonparametric (Spearman) correlation coefficients for the relationship between lipids levels in serum and seminal plasma for 631 subfertile men.

Variable	TG in SP	TC in SP	LDL in SP	HDL in SP
**Serum TG**	0.081^a^	-0.021	0.033	-0.054
**Serum TC**	0.060	-0.038	0.019	-0.045
**Serum LDL**	0.088^a^	-0.027	0.020	0.018
**Serum HDL**	-0.031	-0.002	-0.060	0.006

SP, seminal plasma.

^a^, *P* ≤ 0.05

TG level in SP was related to serum TG and LDL levels (*P* = 0.042 and 0.026).

### Dichotomized analyses for semen parameters

Due to the lack of the correlations between serum lipids levels and semen parameters, while significant correlations between lipids levels in seminal plasma and multiple semen parameters, we further compared the lipids levels in seminal plasma based on the dichotomized analyses for semen parameters, and the results were shown in Table 4. The results indicated that TG, TC, LDL and HDL levels in seminal plasma in patients with oligospermia, asthenospermia and teratozoospermia were higher than those with normal sperm concentration, motility or morphology. Moreover, HDL levels in patients with asthenospermia and teratozoospermia were significantly higher than those with normal sperm motility or morphology (*t* = 2.434, *P* = 0.015; *t* = 2.021, *P* = 0.044).

**Table 4:** Comparisons of lipids levels in seminal plasma based on the dichotomized analyses for semen parameters (mean ± SD).

Variable	*n*	TG in SP (mmol/L)	TC in SP (mmol/L)	HDL in SP (mmol/L)	LDL in SP (mmol/L)
**Sperm concentration**					
** < 15×10**^**6**^**/ml**	81	0.13 ± 0.10	0.87 ± 0.45	0.38 ± 0.18	0.71 ± 0.30
** ≥ 15×10**^**6**^**/ml**	550	0.11 ± 0.09	0.82 ± 0.51	0.37 ± 0.20	0.70 ± 0.32
**Total sperm count**					
** < 39×10**^**6**^**/ejaculate**	58	0.13 ± 0.11	0.91 ± 0.44	0.39 ± 0.16	0.74 ± 0.30
** ≥ 39×10**^**6**^**/ejaculate**	573	0.11 ± 0.09	0.82 ± 0.51	0.37 ± 0.20	0.70 ± 0.32
**Progressive motility**					
** < 32%**	285	0.12 ± 0.10	0.84 ± 0.50	0.39 ± 0.19^a^	0.71 ± 0.30
** ≥ 32%**	346	0.11 ± 0.08	0.81 ± 0.51	0.35 ± 0.21	0.70 ± 0.33
**Normal sperm morphology**					
** < 4%**	183	0.12 ± 0.11	0.84 ± 0.51	0.39 ± 0.20^a^	0.70 ± 0.32
** ≥ 4%**	448	0.11 ± 0.08	0.82 ± 0.50	0.36 ± 0.20	0.69 ± 0.30

SP, seminal plasma.

^a^, *P* ≤ 0.05 *versus* the normal sperm motility or morphology.

### Correlations of semen parameters with age and serum reproductive hormones

Due to the possible effects of age and reproductive hormones on semen parameters, we investigated the correlations of semen parameters with age and serum reproductive hormones. The results were shown in Table 5. We found that there was no correlation between age and semen parameters, but age was positively related to serum FSH level, and negatively related to serum TT and LH levels. Serum LH level was negatively related to PR, normal sperm morphology (NSM) and TNPMS, while serum FSH level was negatively related to sperm concentration (SC), total sperm count (TSC), sperm motility, NSM and TNPMS. Serum SHBG level was positively related to semen volume. However, there was no correlation between serum TT and E2 levels and semen parameters such as semen volume, SC, TSC, PR, sperm motility, NSM and TNPMS.

**Table 5:** Nonparametric (Spearman) correlation coefficients between semen parameters and age and serum reproductive hormones.

Variable	Age	Serum LH	Serum FSH	Serum TT	Serum E2	Serum SHBG
**Age**	-	-0.102^b^	0.130^b^	-0.115^b^	-0.024	0.010
**Semen volume**	-0.012	0.003	0.027	0.015	-0.059	0.085^b^
**SC**	0.038	-0.045	-0.171^a^	0.038	0.005	-0.003
**TSC**	0.044	-0.043	-0.166^a^	0.037	-0.001	0.021
**PR**	-0.048	-0.086^b^	-0.038	0.006	0.019	-0.001
**MOT**	-0.062	-0.069	-0.089^b^	0.021	0.020	-0.006
**NSM**	0.008	-0.116^b^	-0.119^b^	0.001	0.054	-0.053
**TNPMS**	0.016	-0.085^b^	-0.164^a^	0.032	0.028	-0.003

SC, sperm concentration; TSC, total sperm count; PR, progressive motility; MOT, sperm motility; NSM, normal sperm morphology; TNPMS, total normal-progressively motile sperm count.

^a^, *P* ≤ 0.001

^b^, *P* ≤ 0.05

Age was positively related to serum FSH level, but negatively related to serum TT and LH levels. Serum LH level was negatively related to PR, NSM and TNPMS, while serum FSH level was negatively related to SC, TSC, MOT, NSM and TNPMS. Serum SHBG level was positively related to semen volume. However, there was no correlation between serum TT and E2 levels and semen parameters such as semen volume, SC, TSC, PR, MOT, NSM and TNPMS.

### Linear regression analysis of the factors correlated with semen quality

By observing the data from Tables 2 and 5, we found that semen volume was correlated with serum TG and HDL levels, seminal plasma LDL level and serum SHBG level, both of sperm concentration and total sperm count with seminal plasma HDL level and serum FSH level, PR with seminal plasma TG, TC and LDL levels and serum LH level, sperm motility with seminal plasma TC level and serum FFA and FSH levels, normal sperm morphology with serum FSH and LH levels, and TNPMS with seminal plasma TC and serum FSH and LH levels. In order to avoid the interaction of these factors on semen parameters, especially the effects of age and reproductive hormones on semen parameters, we further analyzed the effects of these factors on each of semen parameters after adjusting age and serum LH, FSH, TT, E2 and SHBG levels by linear regression, and the results showed that semen volume was significantly correlated with seminal plasma LDL (*t* = -2.528, *P* = 0.012) and serum TG (*t* = -2.008, *P* = 0.045), both of sperm concentration and total sperm count with seminal plasma HDL (*t* = -2.208, *P* = 0.028; *t* = -3.166, *P* = 0.002), and both of PR and sperm motility with seminal plasma TC (*t* = -2.509, *P* = 0.012; *t* = -1.956, *P* = 0.051).

## Discussion

Numerous studies suggested that obesity and overweight were associated with lower fertility [4]. Animal studies showed that high-fat diet might increase body weight of mice, especially serum TG level, and decrease testosterone level, then result in male infertility [13]. Although controversial results in the relationships between obesity and male fertility exist, some studies suggested that BMI was inversely related to semen volume, sperm concentration, total sperm count, sperm motility and percent of normal sperm morphology [14–15]. Studies on the lipid composition of human testis in patients with bilateral varicocele as cause of infertility showed abundant lipids in human testis tissue, and the total lipids formed 1.90 percent of the total wet weight of the tissue. Testicular cholesterol formed 26.50 percent, glycerides 28.50 percent and phospholipids 45 percent of the total lipids [16]. There were also abundant cholesterol and phospholipids in sperm membrane [17], especially polyunsaturated fatty acids (PUFA) closely related to sperm function [18–22]. During spermatogenesis, sperm maturation, capacitation and acrosome reaction, the lipid composition in sperm membrane changed significantly [23–25], and these changes were possibly based on the transfer of cholesterol and phospholipids between sperm and seminal plasma [26–27]. Several studies showed significant correlation between lipid composition in sperm membrane and seminal plasma [28], and increased phospholipid levels in seminal plasma in oligospermia and azoospermia patients [29]. However, it was unclear about the correlations between lipids levels in serum and seminal plasma, obesity-associated markers and semen parameters.

In present study, we analyzed the correlations between lipids levels in serum and seminal plasma, obesity-associated markers and semen parameters in 631 subfertile men. As expected, serum TC, TG and LDL levels increased with increasing age, BMI, WC, WHtR and WHR, while serum HDL level decreased with increasing BMI, WC, WHtR and WHR, suggesting that obesity could definitely influence on lipid metabolism. However, it was a surprise that serum lipids levels such as TC, TG, LDL and HDL were almost unrelated to semen parameters except only slightly correlations between serum TG and HDL levels and semen volume, and between serum FFA level and sperm motility. This observation was similar to the results reported by Hagiuda and colleagues [30].

Next, we analyzed the correlations of lipids levels in serum and seminal plasma, and the results showed the modest correlations between TG level in seminal plasma and serum TG and LDL levels (*P* = 0.042 and 0.026), suggesting that lipids in seminal plasma might not directly come from blood. The possible origin might be epithelial cells in male reproductive tract. This raises the question about the origin of lipids in seminal plasma and how they are regulated.

Therefore, we further analyzed the correlations of lipids level in seminal plasma with age, obesity-associated markers and semen parameters, and the results showed that TG, TC and LDL levels in seminal plasma increased with age, and only TG level in seminal plasma was positively related to BMI, WC, WHtR and WHR. Unlike lipids levels in serum, TG, TC, LDL and HDL levels in seminal plasma were all negatively related to some of semen parameters. For examples, seminal plasma TG level was negatively related to PR; seminal plasma TC level was negatively related to PR, sperm motility and TNPMS; seminal plasma LDL level was negatively related to semen volume and PR; and seminal plasma HDL level negatively related to sperm concentration and total sperm count. These observations suggested that elevated lipids levels in seminal plasma might have adverse effect on sperm quality. It was worth noting that serum HDL level was negatively related to serum TC, TG and LDL levels, while seminal plasma HDL level was positively related to seminal plasma TC, TG and LDL levels. Unlike serum HDL, seminal plasma HDL seems no protective effects on sperm quality.

In order to further evaluate the adverse effect of seminal plasma lipids on sperm quality, we compared seminal plasma lipids level based on the dichotomized analyses for semen parameters, including sperm concentration, total sperm count, progressive motility and normal sperm morphology, and found that seminal plasma TG, TC, LDL and HDL levels in patients with oligospermia, asthenospermia and teratozoospermia increased when compared with the normal sperm concentration, motility or morphology, especially HDL level significantly higher in patients with asthenospermia and teratozoospermia, suggesting the adverse effect of HDL on sperm quality.

From above-mentioned analysis, we found that elevated lipids levels in seminal plasma might have adverse effect on semen quality. However, it was known that age and reproductive hormones also might have some effects on semen quality. Therefore, we further investigated the correlations of semen parameters with age and serum reproductive hormones, and the correlations of seminal plasma lipids with semen parameters after adjusting age and reproductive hormones. We found that there was no correlation between age, serum TT and E2 levels and semen parameters, but serum LH and FSH levels were negatively related to sperm concentration, total sperm count, sperm motility, PR, normal sperm morphology or TNPMS, which was similar to our previous results [10]. After adjusting age and serum LH, FSH, TT, E2 and SHBG levels, we still found that semen volume was significantly correlated with seminal plasma LDL (*P* = 0.012), both of sperm concentration and total sperm count with seminal plasma HDL (*P* = 0.028 and 0.002), and both of PR and sperm motility with seminal plasma TC (*P* = 0.012 and 0.051), demonstrating that elevated lipids levels in seminal plasma might have adverse effect on semen quality.

However, we recognized that our results were inconsistent with some reports. For example, it was reported that elevated serum TG and LDL levels were associated with poor sperm quality or decreased sperm motility [31–32], and that there was no significant difference in seminal plasma TC levels between fertile and infertile men [33]. It was also suggested that obesity leads to the change of semen quality by the most possible way to affect reproductive hormones [34–35]. The reasons leading to these discrepancies may be attributed to the different study population, the size of samples, and the inaccuracy of the determination results. Our study was based on prospective design, and the results were blindly evaluated. In our study, a relative large size of samples was included, and the stringent exclusion criteria were employed. The analyses for all of semen parameters, serum reproductive hormones, and serum and seminal plasma lipids were performed with strict quality control. In order to avoid potential influence of particles in semen, such as sperm, lecithin body on the level, and the determination method of lipids in seminal plasma, seminal plasma was isolated from semen samples by centrifugation at 12 000 ×g for 5 minutes. The results of preliminary experiments for such seminal plasma samples showed good repeatability (CV< 5%). In previous studies [36–38], we have shown that there were still many spermatozoa in some seminal plasma samples obtained at 3 000 ×g centrifugation for 15 minutes. So, the inconsistency for some previously reported results may be due to the sperm residue in seminal plasma. Moreover, four common-used lipids markers in seminal plasma were comprehensively compared with obesity-associated markers, serum lipids levels and semen parameters, and the possible effects of age and reproductive hormones on the relationship between seminal plasma lipids and semen parameters were adjusted. All these efforts provided a guarantee for drawing meaningful conclusions.

Inevitably, our study had some limitations. Because of the variety of causes leading to male infertility and the differences in races of people, dietary and living environment, our study results might be unsuitable for the western countries. However, according to our results that obesity leaded to increased serum lipids levels, and then increased seminal plasma lipids levels, we deemed that obesity may affect semen quality mainly by abnormal metabolism of lipids in male reproductive system. It is possible that the abnormal lipids levels in male reproductive system are the direct reason leading to decreased semen quality.

## Conclusions

Obesity may suggest abnormal lipids level in serum. However, among four serum lipids markers, only TG level was slightly related to seminal plasma TG level. Serum lipids almost had no correlation with semen parameters, while lipids levels in seminal plasma could influence on semen volume, sperm motility, PR, sperm concentration and total sperm count to a certain extent. The data presented in this study indicated that obesity may affect semen quality mainly by abnormal metabolism of lipids in male reproductive system. It is possible that the increased lipids levels in seminal plasma but not serum are the direct reason leading to decreased semen quality. However, questions about the origin of seminal plasma lipids, the mechanism of lipids affecting sperm quality, and the factors leading to the metabolic disorder of the lipids remain.

## References

[1] Chavarro JE, Toth TL, Wright DL, Meeker JD, Hauser R. Body mass index in relation to semen quality, sperm DNA integrity, and serum reproductive hormone levels among men attending an infertility clinic. Fertil Steril. 2010; 93: 2222–2231. 10.1016/j.fertnstert.2009.01.100 19261274 PMC2864498

[2] MacDonald AA, Herbison GP, Showell M, Farquhar CM. The impact of body mass index on semen parameters and reproductive hormones in human males: a systematic review with meta-analysis. Hum Reprod Update. 2010; 16: 293–311. 10.1093/humupd/dmp047 19889752

[3] Cabler S, Agarwal A, Flint M, du Plessis SS. Obesity: modern man's fertility nemesis. Asian J Androl. 2010; 12: 480–489. 10.1038/aja.2010.38 20531281 PMC3739371

[4] Reverchon M, Maillard V, Froment P, Ramé C, Dupont J. Adiponectin and resistin: a role in the reproductive functions? Med Sci (Paris). 2013; 29: 417–424. 23621938 10.1051/medsci/2013294016

[5] Janssen I, Katzmarzyk PT, Ross R. Waist circumference and not body mass index explains obesity-related health risk. Am J Clin Nutr. 2004; 79: 379–384. 14985210 10.1093/ajcn/79.3.379

[6] Petersson H, Daryani A, Risérus U. Sagittal abdominal diameter as a marker of inflammation and insulin resistance among immigrant women from the Middle East and native Swedish women: a cross-sectional study. Cardiovasc Diabetol. 2007; 6: 10 17391519 10.1186/1475-2840-6-10 PMC1847804

[7] Maqdasy S, Baptissart M, Vega A, Baron S, Lobaccaro JM, Volle DH. Cholesterol and male fertility: what about orphans and adopted? Mol Cell Endocrinol. 2013; 368: 30–46. 10.1016/j.mce.2012.06.011 22766106

[8] Casado ME, Huerta L, Ortiz AI, Pérez-Crespo M, Gutiérrez-Adán A, Kraemer FB, et al HSL-knockout mouse testis exhibits class B scavenger receptor upregulation and disrupted lipid raft microdomains. J Lipid Res. 2012; 53: 2586–2597. 10.1194/jlr.M028076 22988039 PMC3494238

[9] Furland NE, Luquez JM, Oresti GM, Aveldaño MI. Mild testicular hyperthermia transiently increases lipid droplet accumulation and modifies sphingolipid and glycerophospholipid acyl chains in the rat testis. Lipids. 2011; 46: 443–454. 10.1007/s11745-011-3527-3 21318468

[10] Lu JC, Jing J, Dai JY, Zhao AZ, Yao Q, Fan K, et al Body mass index, waist-to-hip ratio, waist circumference and waist-to-height ratio cannot predict male semen quality: a report of 1 231 subfertile Chinese men. Andrologia. 2015; 47: 1047–1054. 10.1111/and.12376 25418484

[11] Lu JC, Huang YF, Lu NQ. Computer-aided sperm analysis (CASA): past, present and future. Andrologia. 2014; 46: 329–338. 10.1111/and.12093 23550608

[12] World Health Organization. Laboratory manual for the examination and processing of human semen. 5th ed Geneva: World Health Organization, 2010.

[13] Erdemir F, Atilgan D, Markoc F, Boztepe O, Suha-Parlaktas B, Sahin S. The effect of diet induced obesity on testicular tissue and serum oxidative stress parameters. Actas Urol Esp. 2012; 36: 153–159. 10.1016/j.acuro.2011.06.019 21959063

[14] Shayeb AG, Harrild K, Mathers E, Bhattacharya S. An exploration of the association between male body mass index and semen quality. Reprod Biomed Online. 2011; 23: 717–723. 10.1016/j.rbmo.2011.07.018 22019618

[15] Martini AC, Tissera A, Estofán D, Molina RI, Mangeaud A, de Cuneo MF, et al Overweight and seminal quality: a study of 794 patients. Fertil Steril. 2010; 94: 1739–1743. 10.1016/j.fertnstert.2009.11.017 20056217

[16] Sheriff DS. The lipid composition of human testis in patients with bilateral varicocele as cause of infertility. Andrologia. 1982; 14: 150–153. 7103134 10.1111/j.1439-0272.1982.tb03117.x

[17] Argov-Argaman N, Mahgrefthe K, Zeron Y, Roth Z. Variation in lipid profiles within semen compartments—the bovine model of aging. Theriogenology. 2013; 80: 712–721. 10.1016/j.theriogenology.2013.05.024 23830232

[18] Casado ME, Pastor O, Mariscal P, Canfrán-Duque A, Martínez-Botas J, Kraemer FB, et al Hormone-sensitive lipase deficiency disturbs the fatty acid composition of mouse testis. Prostaglandins Leukot Essent Fatty Acids. 2013; 88: 227–233. 10.1016/j.plefa.2012.12.005 23369366

[19] Tavilani H, Doosti M, Nourmohammadi I, Mahjub H, Vaisiraygani A, Salimi S, et al Lipid composition of spermatozoa in normozoospermic and asthenozoospermic males. Prostaglandins Leukot Essent Fatty Acids. 2007; 77: 45–50. 17693070 10.1016/j.plefa.2007.07.001

[20] Tavilani H, Doosti M, Abdi K, Vaisiraygani A, Joshaghani HR. Decreased polyunsaturated and increased saturated fatty acid concentration in spermatozoa from asthenozoospermic males as compared with normozoospermic males. Andrologia. 2006; 38: 173–178. 16961570 10.1111/j.1439-0272.2006.00735.x

[21] Aksoy Y, Aksoy H, Altinkaynak K, Aydin HR, Ozkan A. Sperm fatty acid composition in subfertile men. Prostaglandins Leukot Essent Fatty Acids. 2006; 75: 75–79. 16893631 10.1016/j.plefa.2006.06.002

[22] Lenzi A, Gandini L, Lombardo F, Picardo M, Maresca V, Panfili E, et al Polyunsaturated fatty acids of germ cell membranes, glutathione and blutathione-dependent enzyme-PHGPx: from basic to clinic. Contraception. 2002; 65: 301–304. 12020783 10.1016/s0010-7824(02)00276-7

[23] Keber R, Rozman D, Horvat S. Sterols in spermatogenesis and sperm maturation. J Lipid Res. 2013; 54: 20–23. 10.1194/jlr.R032326 23093550 PMC3520525

[24] Nolan JP, Hammerstedt RH. Regulation of membrane stability and the acrosome reaction in mammalian sperm. FASEB J. 1997; 11: 670–682. 9240968 10.1096/fasebj.11.8.9240968

[25] Rana AP, Misra S, Majumder GC, Ghosh A. Phospholipid asymmetry of goat sperm plasma membrane during epididymal maturation. Biochim Biophys Acta. 1993; 1210: 1–7. 8257711 10.1016/0005-2760(93)90041-7

[26] Scolari S, Müller K, Bittman R, Herrmann A, Müller P. Interaction of mammalian seminal plasma protein PDC-109 with cholesterol: implications for a putative CRAC domain. Biochemistry. 2010; 49: 9027–9031. 10.1021/bi101257c 20863067

[27] Masson D, Drouineaud V, Moiroux P, Gautier T, Dautin G, Schneider M, et al Human seminal plasma displays significant phospholipid transfer activity due to the presence of active phospholipid transfer protein. Mol Hum Reprod. 2003; 9: 457–464. 12837922 10.1093/molehr/gag062

[28] Huacuja L, Delgado NM, Calzada L, Wens A, Reyes R, Pedrón N, et al Exchange of lipids between spermatozoa and seminal plasma in normal and pathological human semen. Arch Androl. 1981; 7: 343–349. 7316607 10.3109/01485018108999329

[29] Sebastian SM, Selvaraj S, Aruldhas MM, Govindarajulu P. Pattern of neutral and phospholipids in the semen of normospermic, oligospermic and azoospermic men. J Reprod Fertil. 1987; 79: 373–378. 3572873 10.1530/jrf.0.0790373

[30] Hagiuda J, Ishikawa H, Furuuchi T, Hanawa Y, Marumo K. Relationship between dyslipidaemia and semen quality and serum sex hormone levels: an infertility study of 167 Japanese patients. Andrologia. 2014; 46: 131–135. 10.1111/and.12057 23278423

[31] Ergün A, Köse SK, Aydos K, Ata A, Avci A. Correlation of seminal parameters with serum lipid profile and sex hormones. Arch Androl. 2007; 53: 21–23. 17364460 10.1080/01485010600888961

[32] Ebesunun MO, Solademi BA, Shittu OB, Anetor JI, Onuegbu JA, Olisekodiaka JM, et al Plasma and semen ascorbic levels in spermatogenesis. West Afr J Med. 2004; 23: 290–293. 15730086 10.4314/wajm.v23i4.28143

[33] Meseguer M, Garrido N, Martínez-Conejero JA, Simón C, Pellicer A, Remohí J. Relationship between standard semen parameters, calcium, cholesterol contents, and mitochondrial activity in ejaculated spermatozoa from fertile and infertile males. J Assist Reprod Genet. 2004; 21: 445–451. 15704520 10.1007/s10815-004-8761-7 PMC3455617

[34] Macdonald AA, Stewart AW, Farquhar CM. Body mass index in relation to semen quality and reproductive hormones in New Zealand men: a cross-sectional study in fertility clinics. Hum Reprod. 2013; 28: 3178–3187. 10.1093/humrep/det379 24129611

[35] Sermondade N, Faure C, Fezeu L, Shayeb AG, Bonde JP, Jensen TK, et al BMI in relation to sperm count: an updated systematic review and collaborative meta-analysis. Hum Reprod Update. 2013; 19: 221–231. 10.1093/humupd/dms050 23242914 PMC3621293

[36] Lu JC, Xu HR, Chen F, Huang YF, Lu NQ. Standardization and quality control for the determination of alphaglucosidase in seminal plasma. Arch Androl. 2006; 52: 447–453. 17050326 10.1080/01485010600822705

[37] Lu JC, Chen F, Xu HR, Huang YF, Lu NQ. Preliminary investigations on the standardization and quality control for the determination of acid phosphatase activity in seminal plasma. Clin Chim Acta. 2007; 375: 76–81. 16857182 10.1016/j.cca.2006.06.005

[38] Lu JC, Chen F, Xu HR, Huang YF, Lu NQ. Standardization and quality control for determination of fructose in seminal plasma. J Androl. 2007; 28: 207–213. 17135632 10.2164/jandrol.106.001552

